# Correction: Evaluating cognitive depth of AI-generated multiple-choice questions with Bloom’s Taxonomy

**DOI:** 10.1371/journal.pone.0346800

**Published:** 2026-04-07

**Authors:** Trang Thi Nguyen, Linh Nguyen, Ha Thi Nguyet Do, Huong Thi Thu Nguyen, Son Minh Tong

There are errors in the author affiliations. The correct affiliations are as follows:

Trang Thi Nguyen^1^, Linh Nguyen^2^, Ha Thi Nguyet Do^1^, Huong Thi Thu Nguyen^2^, Son Minh Tong^2^

**1** Faculty of Dentistry, Phenikaa University, Hanoi, Vietnam, **2** School of Dentistry, Hanoi medical university, Hanoi, Vietnam.

The first and second authors, Trang Thi Nguyen and Linh Nguyen, should be noted as shared co-corresponding authors of this work. Trang Thi Nguyen’s email address is: trang.nguyenthi@phenikaa-uni.edu.vn.

## References

[1] Nguyen TT, Nguyen L, Do HTN, Nguyen HTT, Tong SM. Evaluating cognitive depth of AI-generated multiple-choice questions with Bloom’s Taxonomy. PLoS One. 2026;21(2):e0341317. doi: 10.1371/journal.pone.0341317 41758892 PMC12948114

