# Supplementary figures and images for: Evaluating cognitive depth of AI-generated multiple-choice questions with Bloom’s Taxonomy
Source: PLoS One. 2026 Feb 27;21(2):e0341317. doi: 10.1371/journal.pone.0341317 (PMC12948114; doi:10.1371/journal.pone.0341317)

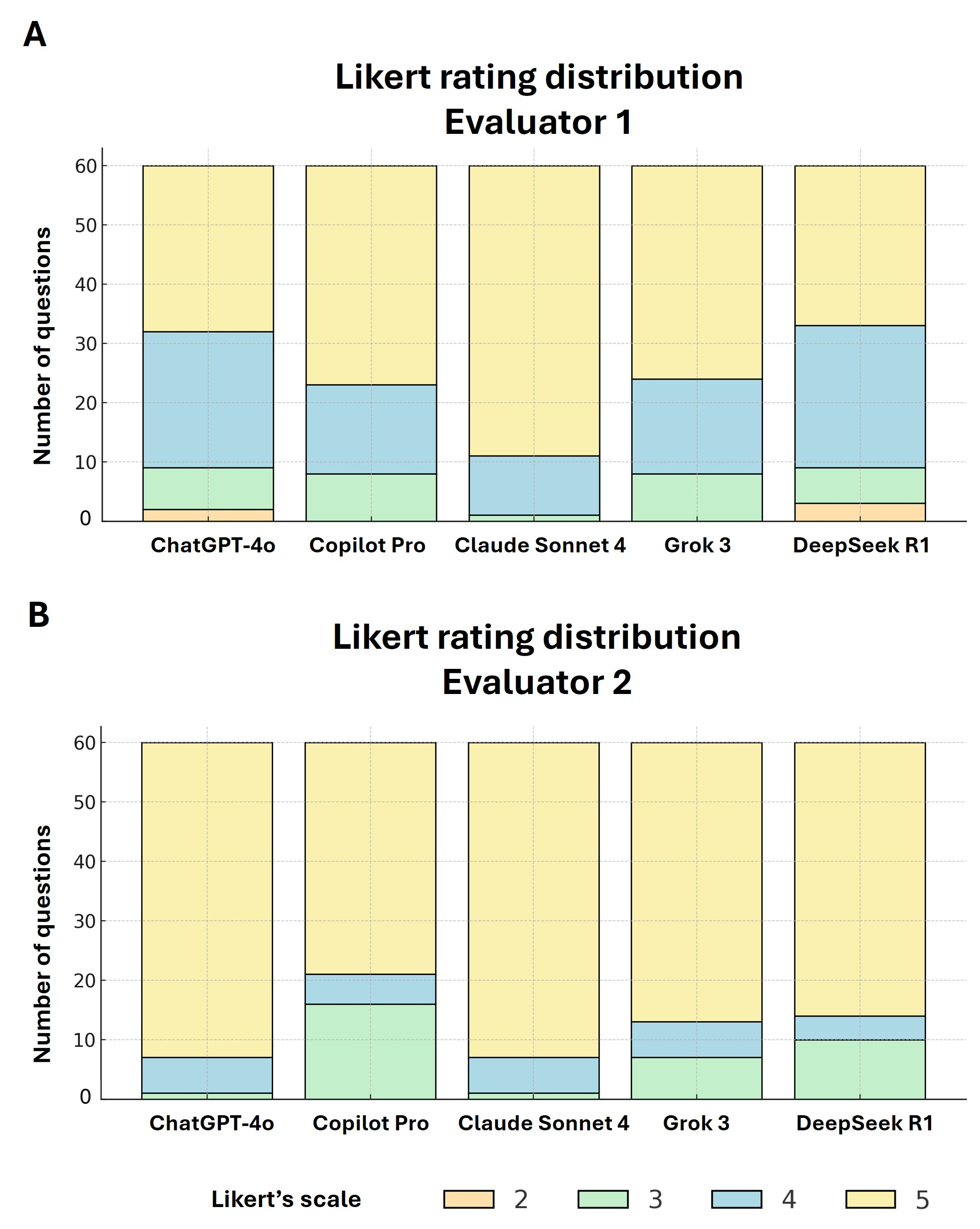

Supplement: S1 Fig — (TIFF) [file pone.0341317.s001.tiff]
