# Supplementary material for: Evaluating cognitive depth of AI-generated multiple-choice questions with Bloom’s Taxonomy
Source: PLoS One. 2026 Feb 27;21(2):e0341317. doi: 10.1371/journal.pone.0341317 (PMC12948114; doi:10.1371/journal.pone.0341317)
Supplement: S2 File — (HTML) [file pone.0341317.s003.html]

boxplot\_heatmap.ipynb


|  |
| --- |
| boxplot\_heatmap.ipynb |

```
{
  "nbformat": 4,
  "nbformat_minor": 0,
  "metadata": {
    "colab": {
      "provenance": [],
      "toc_visible": true
    },
    "kernelspec": {
      "name": "python3",
      "display_name": "Python 3"
    },
    "language_info": {
      "name": "python"
    }
  },
  "cells": [
    {
      "cell_type": "code",
      "source": [
        "!pip install krippendorff"
      ],
      "metadata": {
        "colab": {
          "base_uri": "https://localhost:8080/"
        },
        "id": "yPsQm-ObDZ9u",
        "outputId": "adce29e4-0a50-4c07-81f6-09e616a852bf"
      },
      "execution_count": 1,
      "outputs": [
        {
          "output_type": "stream",
          "name": "stdout",
          "text": [
            "Collecting krippendorff\n",
            "  Downloading krippendorff-0.8.1-py3-none-any.whl.metadata (3.0 kB)\n",
            "Requirement already satisfied: numpy<3,>=1.21 in /usr/local/lib/python3.11/dist-packages (from krippendorff) (2.0.2)\n",
            "Downloading krippendorff-0.8.1-py3-none-any.whl (18 kB)\n",
            "Installing collected packages: krippendorff\n",
            "Successfully installed krippendorff-0.8.1\n"
          ]
        }
      ]
    },
    {
      "cell_type": "code",
      "execution_count": 2,
      "metadata": {
        "colab": {
          "base_uri": "https://localhost:8080/",
          "height": 373
        },
        "id": "o6rQ1GMJWdSL",
        "outputId": "e97bac35-5270-4878-d008-e22f77303615"
      },
      "outputs": [
        {
          "output_type": "error",
          "ename": "FileNotFoundError",
          "evalue": "[Errno 2] No such file or directory: 'Chamdiem_Trang_0621.xlsx'",
          "traceback": [
            "\u001b[0;31m---------------------------------------------------------------------------\u001b[0m",
            "\u001b[0;31mFileNotFoundError\u001b[0m                         Traceback (most recent call last)",
            "\u001b[0;32m/tmp/ipython-input-2-2575030566.py\u001b[0m in \u001b[0;36m<cell line: 0>\u001b[0;34m()\u001b[0m\n\u001b[1;32m      5\u001b[0m \u001b[0;31m# Load your Excel file\u001b[0m\u001b[0;34m\u001b[0m\u001b[0;34m\u001b[0m\u001b[0m\n\u001b[1;32m      6\u001b[0m \u001b[0mfile_path\u001b[0m \u001b[0;34m=\u001b[0m \u001b[0;34m\"Chamdiem_Trang_0621.xlsx\"\u001b[0m  \u001b[0;31m# Replace with your actual file path\u001b[0m\u001b[0;34m\u001b[0m\u001b[0;34m\u001b[0m\u001b[0m\n\u001b[0;32m----> 7\u001b[0;31m \u001b[0mdf\u001b[0m \u001b[0;34m=\u001b[0m \u001b[0mpd\u001b[0m\u001b[0;34m.\u001b[0m\u001b[0mread_excel\u001b[0m\u001b[0;34m(\u001b[0m\u001b[0mfile_path\u001b[0m\u001b[0;34m,\u001b[0m \u001b[0msheet_name\u001b[0m\u001b[0;34m=\u001b[0m\u001b[0;34m\"Sheet1\"\u001b[0m\u001b[0;34m,\u001b[0m \u001b[0mheader\u001b[0m\u001b[0;34m=\u001b[0m\u001b[0;36m1\u001b[0m\u001b[0;34m)\u001b[0m\u001b[0;34m\u001b[0m\u001b[0;34m\u001b[0m\u001b[0m\n\u001b[0m\u001b[1;32m      8\u001b[0m \u001b[0;34m\u001b[0m\u001b[0m\n\u001b[1;32m      9\u001b[0m \u001b[0;31m# Rename for clarity (adjust if needed)\u001b[0m\u001b[0;34m\u001b[0m\u001b[0;34m\u001b[0m\u001b[0m\n",
            "\u001b[0;32m/usr/local/lib/python3.11/dist-packages/pandas/io/excel/_base.py\u001b[0m in \u001b[0;36mread_excel\u001b[0;34m(io, sheet_name, header, names, index_col, usecols, dtype, engine, converters, true_values, false_values, skiprows, nrows, na_values, keep_default_na, na_filter, verbose, parse_dates, date_parser, date_format, thousands, decimal, comment, skipfooter, storage_options, dtype_backend, engine_kwargs)\u001b[0m\n\u001b[1;32m    493\u001b[0m     \u001b[0;32mif\u001b[0m \u001b[0;32mnot\u001b[0m \u001b[0misinstance\u001b[0m\u001b[0;34m(\u001b[0m\u001b[0mio\u001b[0m\u001b[0;34m,\u001b[0m \u001b[0mExcelFile\u001b[0m\u001b[0;34m)\u001b[0m\u001b[0;34m:\u001b[0m\u001b[0;34m\u001b[0m\u001b[0;34m\u001b[0m\u001b[0m\n\u001b[1;32m    494\u001b[0m         \u001b[0mshould_close\u001b[0m \u001b[0;34m=\u001b[0m \u001b[0;32mTrue\u001b[0m\u001b[0;34m\u001b[0m\u001b[0;34m\u001b[0m\u001b[0m\n\u001b[0;32m--> 495\u001b[0;31m         io = ExcelFile(\n\u001b[0m\u001b[1;32m    496\u001b[0m             \u001b[0mio\u001b[0m\u001b[0;34m,\u001b[0m\u001b[0;34m\u001b[0m\u001b[0;34m\u001b[0m\u001b[0m\n\u001b[1;32m    497\u001b[0m             \u001b[0mstorage_options\u001b[0m\u001b[0;34m=\u001b[0m\u001b[0mstorage_options\u001b[0m\u001b[0;34m,\u001b[0m\u001b[0;34m\u001b[0m\u001b[0;34m\u001b[0m\u001b[0m\n",
            "\u001b[0;32m/usr/local/lib/python3.11/dist-packages/pandas/io/excel/_base.py\u001b[0m in \u001b[0;36m__init__\u001b[0;34m(self, path_or_buffer, engine, storage_options, engine_kwargs)\u001b[0m\n\u001b[1;32m   1548\u001b[0m                 \u001b[0mext\u001b[0m \u001b[0;34m=\u001b[0m \u001b[0;34m\"xls\"\u001b[0m\u001b[0;34m\u001b[0m\u001b[0;34m\u001b[0m\u001b[0m\n\u001b[1;32m   1549\u001b[0m             \u001b[0;32melse\u001b[0m\u001b[0;34m:\u001b[0m\u001b[0;34m\u001b[0m\u001b[0;34m\u001b[0m\u001b[0m\n\u001b[0;32m-> 1550\u001b[0;31m                 ext = inspect_excel_format(\n\u001b[0m\u001b[1;32m   1551\u001b[0m                     \u001b[0mcontent_or_path\u001b[0m\u001b[0;34m=\u001b[0m\u001b[0mpath_or_buffer\u001b[0m\u001b[0;34m,\u001b[0m \u001b[0mstorage_options\u001b[0m\u001b[0;34m=\u001b[0m\u001b[0mstorage_options\u001b[0m\u001b[0;34m\u001b[0m\u001b[0;34m\u001b[0m\u001b[0m\n\u001b[1;32m   1552\u001b[0m                 )\n",
            "\u001b[0;32m/usr/local/lib/python3.11/dist-packages/pandas/io/excel/_base.py\u001b[0m in \u001b[0;36minspect_excel_format\u001b[0;34m(content_or_path, storage_options)\u001b[0m\n\u001b[1;32m   1400\u001b[0m         \u001b[0mcontent_or_path\u001b[0m \u001b[0;34m=\u001b[0m \u001b[0mBytesIO\u001b[0m\u001b[0;34m(\u001b[0m\u001b[0mcontent_or_path\u001b[0m\u001b[0;34m)\u001b[0m\u001b[0;34m\u001b[0m\u001b[0;34m\u001b[0m\u001b[0m\n\u001b[1;32m   1401\u001b[0m \u001b[0;34m\u001b[0m\u001b[0m\n\u001b[0;32m-> 1402\u001b[0;31m     with get_handle(\n\u001b[0m\u001b[1;32m   1403\u001b[0m         \u001b[0mcontent_or_path\u001b[0m\u001b[0;34m,\u001b[0m \u001b[0;34m\"rb\"\u001b[0m\u001b[0;34m,\u001b[0m \u001b[0mstorage_options\u001b[0m\u001b[0;34m=\u001b[0m\u001b[0mstorage_options\u001b[0m\u001b[0;34m,\u001b[0m \u001b[0mis_text\u001b[0m\u001b[0;34m=\u001b[0m\u001b[0;32mFalse\u001b[0m\u001b[0;34m\u001b[0m\u001b[0;34m\u001b[0m\u001b[0m\n\u001b[1;32m   1404\u001b[0m     ) as handle:\n",
            "\u001b[0;32m/usr/local/lib/python3.11/dist-packages/pandas/io/common.py\u001b[0m in \u001b[0;36mget_handle\u001b[0;34m(path_or_buf, mode, encoding, compression, memory_map, is_text, errors, storage_options)\u001b[0m\n\u001b[1;32m    880\u001b[0m         \u001b[0;32melse\u001b[0m\u001b[0;34m:\u001b[0m\u001b[0;34m\u001b[0m\u001b[0;34m\u001b[0m\u001b[0m\n\u001b[1;32m    881\u001b[0m             \u001b[0;31m# Binary mode\u001b[0m\u001b[0;34m\u001b[0m\u001b[0;34m\u001b[0m\u001b[0m\n\u001b[0;32m--> 882\u001b[0;31m             \u001b[0mhandle\u001b[0m \u001b[0;34m=\u001b[0m \u001b[0mopen\u001b[0m\u001b[0;34m(\u001b[0m\u001b[0mhandle\u001b[0m\u001b[0;34m,\u001b[0m \u001b[0mioargs\u001b[0m\u001b[0;34m.\u001b[0m\u001b[0mmode\u001b[0m\u001b[0;34m)\u001b[0m\u001b[0;34m\u001b[0m\u001b[0;34m\u001b[0m\u001b[0m\n\u001b[0m\u001b[1;32m    883\u001b[0m         \u001b[0mhandles\u001b[0m\u001b[0;34m.\u001b[0m\u001b[0mappend\u001b[0m\u001b[0;34m(\u001b[0m\u001b[0mhandle\u001b[0m\u001b[0;34m)\u001b[0m\u001b[0;34m\u001b[0m\u001b[0;34m\u001b[0m\u001b[0m\n\u001b[1;32m    884\u001b[0m \u001b[0;34m\u001b[0m\u001b[0m\n",
            "\u001b[0;31mFileNotFoundError\u001b[0m: [Errno 2] No such file or directory: 'Chamdiem_Trang_0621.xlsx'"
          ]
        }
      ],
      "source": [
        "import pandas as pd\n",
        "import numpy as np\n",
        "import krippendorff\n",
        "\n",
        "# Load your Excel file\n",
        "file_path = \"Chamdiem_Trang_0621.xlsx\"  # Replace with your actual file path\n",
        "df = pd.read_excel(file_path, sheet_name=\"Sheet1\", header=1)\n",
        "\n",
        "# Rename for clarity (adjust if needed)\n",
        "df = df.rename(columns={'Bloom': 'Trang', 'Bloom.1': 'Linh'})\n",
        "\n",
        "# Drop rows with missing values\n",
        "df_clean = df[['Trang', 'Linh']].dropna()\n",
        "\n",
        "# Prepare the rating matrix: rows = raters, columns = items\n",
        "ratings = np.array([df_clean['Trang'].values, df_clean['Linh'].values])\n",
        "\n",
        "# Compute Krippendorff's alpha (ordinal distance)\n",
        "alpha_ordinal = krippendorff.alpha(reliability_data=ratings, level_of_measurement='ordinal')\n",
        "\n",
        "print(f\"Krippendorff’s Alpha (ordinal): {alpha_ordinal:.3f}\")"
      ]
    },
    {
      "cell_type": "code",
      "source": [
        "import os\n",
        "os.listdir()\n",
        "``>\n",
        "\n",
        "You should see a list of uploaded files, including something like:"
      ],
      "metadata": {
        "id": "T6GGoxWaE95I"
      },
      "execution_count": null,
      "outputs": []
    },
    {
      "cell_type": "code",
      "source": [
        "!pip install krippendorff\n",
        "\n",
        "import pandas as pd\n",
        "import numpy as np\n",
        "import krippendorff\n",
        "\n",
        "# Load the Excel file\n",
        "df = pd.read_excel(\"Chamdiem_Trang_0621.xlsx\", sheet_name=\"Sheet1\", header=1)\n",
        "\n",
        "# Rename for clarity\n",
        "df = df.rename(columns={'Bloom': 'Trang', 'Bloom.1': 'Linh'})\n",
        "\n",
        "# Drop rows with missing values\n",
        "df_clean = df[['Trang', 'Linh']].dropna()\n",
        "\n",
        "# Prepare the rating matrix: rows = raters, columns = items\n",
        "ratings = np.array([df_clean['Trang'].values, df_clean['Linh'].values])\n",
        "\n",
        "# Compute Krippendorff's Alpha (ordinal)\n",
        "alpha = krippendorff.alpha(reliability_data=ratings, level_of_measurement='ordinal')\n",
        "print(f\"Krippendorff’s Alpha (ordinal): {alpha:.3f}\")"
      ],
      "metadata": {
        "id": "cDXX3NhQFG5Y"
      },
      "execution_count": null,
      "outputs": []
    },
    {
      "cell_type": "code",
      "source": [
        "from google.colab import files\n",
        "uploaded = files.upload()"
      ],
      "metadata": {
        "id": "aIJfz3ZnF2x6"
      },
      "execution_count": null,
      "outputs": []
    },
    {
      "cell_type": "code",
      "source": [
        "import os\n",
        "print(os.listdir())"
      ],
      "metadata": {
        "id": "6AF2fmYiF-Ow"
      },
      "execution_count": null,
      "outputs": []
    },
    {
      "cell_type": "code",
      "source": [
        "['Chamdiem_Trang_0621.xlsx']"
      ],
      "metadata": {
        "id": "J-cR7UpOGAGw"
      },
      "execution_count": null,
      "outputs": []
    },
    {
      "cell_type": "code",
      "source": [
        "!pip install krippendorff\n",
        "\n",
        "import pandas as pd\n",
        "import numpy as np\n",
        "import krippendorff\n",
        "\n",
        "df = pd.read_excel(\"Chamdiem_Trang_0621.xlsx\", sheet_name=\"Sheet1\", header=1)\n",
        "df = df.rename(columns={'Bloom': 'Trang', 'Bloom.1': 'Linh'})\n",
        "df_clean = df[['Trang', 'Linh']].dropna()\n",
        "\n",
        "ratings = np.array([df_clean['Trang'].values, df_clean['Linh'].values])\n",
        "\n",
        "alpha = krippendorff.alpha(reliability_data=ratings, level_of_measurement='ordinal')\n",
        "print(f\"Krippendorff’s Alpha (ordinal): {alpha:.3f}\")"
      ],
      "metadata": {
        "id": "0CawLk8OGGP7"
      },
      "execution_count": null,
      "outputs": []
    },
    {
      "cell_type": "code",
      "source": [
        "!pip install krippendorff\n",
        "\n",
        "import pandas as pd\n",
        "import numpy as np\n",
        "import krippendorff\n",
        "\n",
        "# Create the dataset manually (you can also load it from Excel)\n",
        "data = {\n",
        "    \"Trang\": [5, 4, 5, 5, 5, 5, 5, 5, 3, 5, 5, 5, 4, 5, 5, 5, 5, 5,\n",
        "              5, 5, 5, 5, 4, 5, 5, 5, 5, 5, 4, 5, 5, 5, 5, 5, 3,\n",
        "              5, 5, 5, 5, 5, 5, 5, 5, 4, 5, 5, 5, 5, 5, 5, 5, 4, 5,\n",
        "              5, 5, 5, 5, 5, 5, 4, 5, 5, 5, 5, 5, 5, 5, 5, 5, 5, 5,\n",
        "              5, 5, 4, 4, 5, 4, 5, 5, 5, 5, 5],\n",
        "    \"Linh\":  [5, 5, 5, 5, 5, 5, 5, 5, 5, 5, 5, 5, 5, 5, 5, 5, 5, 5,\n",
        "              5, 5, 5, 5, 3, 3, 5, 5, 5, 5, 5, 5, 5, 5, 5, 5, 3,\n",
        "              5, 5, 5, 5, 5, 5, 5, 5, 5, 5, 5, 5, 5, 5, 5, 5, 5, 5,\n",
        "              5, 5, 5, 5, 5, 5, 5, 5, 5, 5, 5, 5, 5, 5, 5, 5, 5, 5,\n",
        "              5, 5, 5, 5, 5, 5, 5, 5, 5, 5, 5]\n",
        "}\n",
        "\n",
        "df = pd.DataFrame(data)\n",
        "\n",
        "# Build rating matrix\n",
        "ratings = np.array([df['Trang'].values, df['Linh'].values])\n",
        "\n",
        "# Calculate Krippendorff's alpha for ordinal data\n",
        "alpha = krippendorff.alpha(reliability_data=ratings, level_of_measurement='ordinal')\n",
        "print(f\"Krippendorff’s Alpha (ordinal): {alpha:.3f}\")"
      ],
      "metadata": {
        "id": "OfqiJpVpJ0xt"
      },
      "execution_count": null,
      "outputs": []
    },
    {
      "cell_type": "code",
      "source": [
        "# Install scikit-learn (if not already installed)\n",
        "!pip install scikit-learn\n",
        "\n",
        "# Import required library\n",
        "from sklearn.metrics import cohen_kappa_score\n",
        "\n",
        "# Example data: Replace these lists with your actual scores\n",
        "rater_trang = [5, 4, 5, 5, 5, 5, 5, 5, 3, 5, 5, 5]\n",
        "rater_linh  = [5, 5, 5, 5, 5, 5, 5, 5, 5, 5, 5, 5]\n",
        "\n",
        "# Calculate weighted Cohen's Kappa using quadratic weights\n",
        "kappa = cohen_kappa_score(rater_trang, rater_linh, weights='quadratic')\n",
        "\n",
        "# Print result\n",
        "print(f\"Weighted Cohen's Kappa (quadratic): {kappa:.3f}\")"
      ],
      "metadata": {
        "id": "kLK7n6hDLFlC"
      },
      "execution_count": null,
      "outputs": []
    },
    {
      "cell_type": "code",
      "source": [
        "import pandas as pd\n",
        "import seaborn as sns\n",
        "import matplotlib.pyplot as plt\n",
        "\n",
        "# Load data\n",
        "df = pd.read_excel(\"Chamdiem_Trang_0621.xlsx\", sheet_name=\"Sheet1\", header=1)\n",
        "\n",
        "# Rename and clean columns\n",
        "df = df.rename(columns={\"Trang\": \"Evaluator 1\", \"Linh\": \"Evaluator 2\", \"LLMs\": \"LLM\"})\n",
        "df = df[[\"LLM\", \"Evaluator 1\", \"Evaluator 2\"]].dropna()\n",
        "\n",
        "# Map LLM codes to names\n",
        "llm_name_map = {\n",
        "    1: \"ChatGPT-4o\",\n",
        "    2: \"Copilot Pro\",\n",
        "    3: \"Claude 4 Sonnet\",\n",
        "    4: \"Grok 3\",\n",
        "    5: \"Deepseek R1\"\n",
        "}\n",
        "df[\"LLM_Name\"] = df[\"LLM\"].map(llm_name_map)\n",
        "\n",
        "# Melt for seaborn\n",
        "summary_stats = df.melt(id_vars=\"LLM_Name\", value_vars=[\"Evaluator 1\", \"Evaluator 2\"])\n",
        "\n",
        "# Calculate mean and median for overlay\n",
        "mean_values = summary_stats.groupby([\"LLM_Name\", \"variable\"])[\"value\"].mean().reset_index()\n",
        "median_values = summary_stats.groupby([\"LLM_Name\", \"variable\"])[\"value\"].median().reset_index()\n",
        "\n",
        "llm_order = [\"ChatGPT-4o\", \"Copilot Pro\", \"Claude 4 Sonnet\", \"Grok 3\", \"Deepseek R1\"]\n",
        "evaluator_offsets = {\"Evaluator 1\": -0.15, \"Evaluator 2\": 0.15}\n",
        "evaluator_colors = {\"Evaluator 1\": \"green\", \"Evaluator 2\": \"purple\"}\n",
        "\n",
        "# Plot\n",
        "plt.figure(figsize=(16, 6))\n",
        "\n",
        "# Base boxplot in gray\n",
        "sns.boxplot(data=summary_stats, x='LLM_Name', y='value', color='lightgray', showcaps=True, fliersize=0)\n",
        "\n",
        "# Add individual x-shaped scores\n",
        "for evaluator in evaluator_colors:\n",
        "    subset = summary_stats[summary_stats['variable'] == evaluator]\n",
        "    sns.stripplot(data=subset, x='LLM_Name', y='value',\n",
        "                  dodge=True, alpha=0.6, color=evaluator_colors[evaluator],\n",
        "                  jitter=True, marker='x', linewidth=1.2, size=7, label=f'{evaluator} score')\n",
        "\n",
        "# Add mean and median markers\n",
        "for _, row in mean_values.iterrows():\n",
        "    x_pos = llm_order.index(row['LLM_Name']) + evaluator_offsets[row['variable']]\n",
        "    plt.plot(x_pos, row['value'], 'D', color='red', markersize=10,\n",
        "             label='Mean' if row['LLM_Name'] == 'ChatGPT-4o' and row['variable'] == 'Evaluator 1' else \"\")\n",
        "for _, row in median_values.iterrows():\n",
        "    x_pos = llm_order.index(row['LLM_Name']) + evaluator_offsets[row['variable']]\n",
        "    plt.plot(x_pos, row['value'], 's', color='blue', markersize=10,\n",
        "             label='Median' if row['LLM_Name'] == 'ChatGPT-4o' and row['variable'] == 'Evaluator 1' else \"\")\n",
        "\n",
        "# Final formatting\n",
        "plt.title(\"Box Plot with Evaluator Scores (x), Mean (◇), and Median (□)\", fontsize=16, weight='bold')\n",
        "plt.xlabel(\"LLMs\", fontsize=20, weight='bold')\n",
        "plt.ylabel(\"Score\", fontsize=16)\n",
        "plt.xticks(fontsize=16, weight='bold')\n",
        "plt.yticks(fontsize=14)\n",
        "plt.ylim(0, 6)\n",
        "\n",
        "# Clean legend\n",
        "handles, labels = plt.gca().get_legend_handles_labels()\n",
        "by_label = dict(zip(labels, handles))\n",
        "plt.legend(by_label.values(), by_label.keys(), bbox_to_anchor=(1.05, 1), loc='upper left', title=\"Legend\", fontsize=12)\n",
        "\n",
        "plt.tight_layout()\n",
        "plt.show()"
      ],
      "metadata": {
        "id": "PsZ1y_YGLLdX"
      },
      "execution_count": null,
      "outputs": []
    },
    {
      "cell_type": "code",
      "source": [
        "import pandas as pd\n",
        "import seaborn as sns\n",
        "import matplotlib.pyplot as plt\n",
        "\n",
        "# Load the Excel file (upload via Google Colab if needed)\n",
        "df = pd.read_excel(\"Chamdiem_Trang_0621.xlsx\", sheet_name=\"Sheet1\", header=1)\n",
        "\n",
        "# Rename columns based on inspection\n",
        "df_scores = df[['Unnamed: 1', 'Bloom', 'Bloom.1']].dropna()\n",
        "df_scores.columns = ['LLM', 'Evaluator 1', 'Evaluator 2']\n",
        "\n",
        "# Map LLM codes to model names\n",
        "llm_name_map = {\n",
        "    1: \"ChatGPT-4o\",\n",
        "    2: \"Copilot Pro\",\n",
        "    3: \"Claude 4 Sonnet\",\n",
        "    4: \"Grok 3\",\n",
        "    5: \"Deepseek R1\"\n",
        "}\n",
        "df_scores[\"LLM_Name\"] = df_scores[\"LLM\"].map(llm_name_map)\n",
        "\n",
        "# Convert to long format for seaborn\n",
        "df_long = df_scores.melt(id_vars=\"LLM_Name\", value_vars=[\"Evaluator 1\", \"Evaluator 2\"],\n",
        "                         var_name=\"Evaluator\", value_name=\"Score\")\n",
        "\n",
        "# Set visual parameters\n",
        "llm_order = [\"ChatGPT-4o\", \"Copilot Pro\", \"Claude 4 Sonnet\", \"Grok 3\", \"Deepseek R1\"]\n",
        "evaluator_offsets = {\"Evaluator 1\": -0.15, \"Evaluator 2\": 0.15}\n",
        "evaluator_colors = {\"Evaluator 1\": \"green\", \"Evaluator 2\": \"purple\"}\n",
        "\n",
        "# Calculate means and medians\n",
        "mean_vals = df_long.groupby([\"LLM_Name\", \"Evaluator\"])[\"Score\"].mean().reset_index()\n",
        "median_vals = df_long.groupby([\"LLM_Name\", \"Evaluator\"])[\"Score\"].median().reset_index()\n",
        "\n",
        "# Initialize plot\n",
        "plt.figure(figsize=(16, 6))\n",
        "sns.boxplot(data=df_long, x=\"LLM_Name\", y=\"Score\", color=\"lightgray\", fliersize=0)\n",
        "\n",
        "# Add evaluator scores with \"x\"\n",
        "for evaluator in evaluator_colors:\n",
        "    data_subset = df_long[df_long[\"Evaluator\"] == evaluator]\n",
        "    sns.stripplot(data=data_subset, x=\"LLM_Name\", y=\"Score\", jitter=True,\n",
        "                  color=evaluator_colors[evaluator], marker='x', linewidth=1.2, size=7,\n",
        "                  label=f\"{evaluator} score\")\n",
        "\n",
        "# Add mean (◇) and median (□)\n",
        "for _, row in mean_vals.iterrows():\n",
        "    x = llm_order.index(row[\"LLM_Name\"]) + evaluator_offsets[row[\"Evaluator\"]]\n",
        "    plt.plot(x, row[\"Score\"], marker=\"D\", color=\"red\", markersize=10,\n",
        "             label=\"Mean\" if row[\"LLM_Name\"] == \"ChatGPT-4o\" and row[\"Evaluator\"] == \"Evaluator 1\" else \"\")\n",
        "\n",
        "for _, row in median_vals.iterrows():\n",
        "    x = llm_order.index(row[\"LLM_Name\"]) + evaluator_offsets[row[\"Evaluator\"]]\n",
        "    plt.plot(x, row[\"Score\"], marker=\"s\", color=\"blue\", markersize=10,\n",
        "             label=\"Median\" if row[\"LLM_Name\"] == \"ChatGPT-4o\" and row[\"Evaluator\"] == \"Evaluator 1\" else \"\")\n",
        "\n",
        "# Format axes and title\n",
        "plt.title(\"Box Plot with Evaluator Scores (x), Mean (◇), and Median (□)\", fontsize=16, weight='bold')\n",
        "plt.xlabel(\"LLMs\", fontsize=20, weight='bold')\n",
        "plt.ylabel(\"Score\", fontsize=16)\n",
        "plt.xticks(fontsize=16, weight='bold')\n",
        "plt.yticks(fontsize=14)\n",
        "plt.ylim(0, 6)\n",
        "\n",
        "# Custom legend\n",
        "handles, labels = plt.gca().get_legend_handles_labels()\n",
        "by_label = dict(zip(labels, handles))\n",
        "plt.legend(by_label.values(), by_label.keys(), bbox_to_anchor=(1.02, 1), loc=\"upper left\", fontsize=12, title=\"Legend\")\n",
        "\n",
        "plt.tight_layout()\n",
        "plt.show()"
      ],
      "metadata": {
        "id": "71qUq31Y_24F"
      },
      "execution_count": null,
      "outputs": []
    },
    {
      "cell_type": "code",
      "source": [
        "import pandas as pd\n",
        "import matplotlib.pyplot as plt\n",
        "import seaborn as sns\n",
        "\n",
        "# Load your data\n",
        "file_path = \"Remember_Level_Data.xlsx\"  # Replace with your actual file\n",
        "df = pd.read_excel(file_path)\n",
        "\n",
        "# Map LLM codes to names\n",
        "llm_name_map = {\n",
        "    1: \"ChatGPT-4o\",\n",
        "    2: \"Copilot Pro\",\n",
        "    3: \"Claude Sonnet 4\",  # Updated name here\n",
        "    4: \"Grok 3\",\n",
        "    5: \"Deepseek R1\"\n",
        "}\n",
        "\n",
        "df['LLM_Name'] = df['LLMs'].map(llm_name_map)\n",
        "\n",
        "# Average the two evaluations\n",
        "df['Mean_Score'] = df[['Trang Bloom', 'Linh Bloom']].mean(axis=1)\n",
        "\n",
        "# Set up the plot\n",
        "plt.figure(figsize=(12, 6))\n",
        "sns.set(style=\"whitegrid\")\n",
        "\n",
        "# Create violin plot with inner box and outer kernel density\n",
        "ax = sns.violinplot(\n",
        "    x='LLM_Name',\n",
        "    y='Mean_Score',\n",
        "    data=df,\n",
        "    inner='box',\n",
        "    linewidth=1.5,\n",
        "    scale='width'\n",
        ")\n",
        "\n",
        "# Add mean (diamond) and median (square)\n",
        "grouped = df.groupby('LLM_Name')['Mean_Score']\n",
        "positions = range(len(grouped))\n",
        "\n",
        "for pos, (name, group) in zip(positions, grouped):\n",
        "    mean_val = group.mean()\n",
        "    median_val = group.median()\n",
        "    plt.plot(pos, mean_val, marker='D', color='blue', markersize=10, label='Mean' if pos == 0 else \"\")\n",
        "    plt.plot(pos, median_val, marker='s', color='red', markersize=10, label='Median' if pos == 0 else \"\")\n",
        "\n",
        "# Final touches\n",
        "plt.title(\"Violin Plot with Full Boxplot, Mean (◇), and Median (□)\", fontsize=14, weight='bold')\n",
        "plt.xlabel(\"LLMs\", fontsize=12)\n",
        "plt.ylabel(\"Score\", fontsize=12)\n",
        "plt.ylim(0, 6)\n",
        "plt.legend(title=\"Markers\")\n",
        "plt.tight_layout()\n",
        "plt.show()"
      ],
      "metadata": {
        "id": "64nsbHyrJFb6"
      },
      "execution_count": null,
      "outputs": []
    },
    {
      "cell_type": "code",
      "source": [
        "import pandas as pd\n",
        "import matplotlib.pyplot as plt\n",
        "import seaborn as sns\n",
        "\n",
        "\n",
        "\n",
        "# Rename columns for clarity\n",
        "df = df.rename(columns={\"Trang\": \"Evaluator 1\", \"Linh\": \"Evaluator 2\", \"LLMs\": \"LLM\"})\n",
        "\n",
        "# Clean data: drop missing values\n",
        "df_clean = df[[\"LLM\", \"Evaluator 1\", \"Evaluator 2\"]].dropna()\n",
        "\n",
        "# Plot the violin plot\n",
        "plt.figure(figsize=(10, 6))\n",
        "sns.violinplot(data=df_clean, x='LLM', y='Evaluator 1', inner=None, color=\"lightblue\")\n",
        "sns.violinplot(data=df_clean, x='LLM', y='Evaluator 2', inner=None, color=\"lightgreen\")\n",
        "\n",
        "# Customize the plot\n",
        "plt.ylim(0, 5)  # Cap y-axis at 5\n",
        "plt.xticks(rotation=45, ha=\"right\", fontsize=12, fontweight='bold')  # LLMs titles bigger and bold\n",
        "plt.yticks([])  # Remove y-axis numeric values\n",
        "plt.title('Violin Plot of Evaluator Scores by LLM', fontsize=16, fontweight='bold')\n",
        "\n",
        "# Show mean and median markers\n",
        "for i, llm in enumerate(df['LLM'].unique()):\n",
        "    eval_1 = df_clean[df_clean['LLM'] == llm]['Evaluator 1']\n",
        "    eval_2 = df_clean[df_clean['LLM'] == llm]['Evaluator 2']\n",
        "    plt.plot([i-0.2, i+0.2], [eval_1.mean(), eval_1.mean()], color=\"darkblue\", lw=2)\n",
        "    plt.plot([i-0.2, i+0.2], [eval_2.mean(), eval_2.mean()], color=\"darkgreen\", lw=2)\n",
        "    plt.scatter([i-0.2], [eval_1.median()], color=\"black\", zorder=5, s=80, label=\"Evaluator 1 Median\" if i == 0 else \"\")\n",
        "    plt.scatter([i+0.2], [eval_2.median()], color=\"black\", zorder=5, s=80, label=\"Evaluator 2 Median\" if i == 0 else \"\")\n",
        "\n",
        "# Display plot\n",
        "plt.legend(loc=\"upper right\")\n",
        "plt.tight_layout()\n",
        "plt.show()"
      ],
      "metadata": {
        "id": "XEub5jbuKmSz"
      },
      "execution_count": null,
      "outputs": []
    },
    {
      "cell_type": "code",
      "source": [],
      "metadata": {
        "id": "wg5pcLorNn6L"
      },
      "execution_count": null,
      "outputs": []
    },
    {
      "cell_type": "code",
      "source": [
        "import pandas as pd\n",
        "import seaborn as sns\n",
        "import matplotlib.pyplot as plt\n",
        "\n",
        "# Load the Excel file (upload via Google Colab if needed)\n",
        "df = pd.read_excel(\"Chamdiem_Trang_0621.xlsx\", sheet_name=\"Sheet1\", header=1)\n",
        "\n",
        "# Rename columns based on inspection\n",
        "df_scores = df[['Unnamed: 1', 'Bloom', 'Bloom.1']].dropna()\n",
        "df_scores.columns = ['LLM', 'Evaluator 1', 'Evaluator 2']\n",
        "\n",
        "# Map LLM codes to model names\n",
        "llm_name_map = {\n",
        "    1: \"ChatGPT-4o\",\n",
        "    2: \"Copilot Pro\",\n",
        "    3: \"Claude 4 Sonnet\",\n",
        "    4: \"Grok 3\",\n",
        "    5: \"Deepseek R1\"\n",
        "}\n",
        "df_scores[\"LLM_Name\"] = df_scores[\"LLM\"].map(llm_name_map)\n",
        "\n",
        "# Convert to long format for seaborn\n",
        "df_long = df_scores.melt(id_vars=\"LLM_Name\", value_vars=[\"Evaluator 1\", \"Evaluator 2\"],\n",
        "                         var_name=\"Evaluator\", value_name=\"Score\")\n",
        "\n",
        "# Set visual parameters\n",
        "llm_order = [\"ChatGPT-4o\", \"Copilot Pro\", \"Claude 4 Sonnet\", \"Grok 3\", \"Deepseek R1\"]\n",
        "evaluator_offsets = {\"Evaluator 1\": -0.15, \"Evaluator 2\": 0.15}\n",
        "evaluator_colors = {\"Evaluator 1\": \"green\", \"Evaluator 2\": \"purple\"}\n",
        "\n",
        "# Calculate means and medians\n",
        "mean_vals = df_long.groupby([\"LLM_Name\", \"Evaluator\"])[\"Score\"].mean().reset_index()\n",
        "median_vals = df_long.groupby([\"LLM_Name\", \"Evaluator\"])[\"Score\"].median().reset_index()\n",
        "\n",
        "# Initialize plot\n",
        "plt.figure(figsize=(16, 6))\n",
        "sns.boxplot(data=df_long, x=\"LLM_Name\", y=\"Score\", color=\"lightgray\", fliersize=0)\n",
        "\n",
        "# Add evaluator scores with \"x\"\n",
        "for evaluator in evaluator_colors:\n",
        "    data_subset = df_long[df_long[\"Evaluator\"] == evaluator]\n",
        "    sns.stripplot(data=data_subset, x=\"LLM_Name\", y=\"Score\", jitter=True,\n",
        "                  color=evaluator_colors[evaluator], marker='x', linewidth=1.2, size=7,\n",
        "                  label=f\"{evaluator} score\")\n",
        "\n",
        "# Add mean (◇) and median (□)\n",
        "for _, row in mean_vals.iterrows():\n",
        "    x = llm_order.index(row[\"LLM_Name\"]) + evaluator_offsets[row[\"Evaluator\"]]\n",
        "    plt.plot(x, row[\"Score\"], marker=\"D\", color=\"red\", markersize=10,\n",
        "             label=\"Mean\" if row[\"LLM_Name\"] == \"ChatGPT-4o\" and row[\"Evaluator\"] == \"Evaluator 1\" else \"\")\n",
        "\n",
        "for _, row in median_vals.iterrows():\n",
        "    x = llm_order.index(row[\"LLM_Name\"]) + evaluator_offsets[row[\"Evaluator\"]]\n",
        "    plt.plot(x, row[\"Score\"], marker=\"s\", color=\"blue\", markersize=10,\n",
        "             label=\"Median\" if row[\"LLM_Name\"] == \"ChatGPT-4o\" and row[\"Evaluator\"] == \"Evaluator 1\" else \"\")\n",
        "\n",
        "# Format axes and title\n",
        "plt.title(\"Box Plot with Evaluator Scores (x), Mean (◇), and Median (□)\", fontsize=16, weight='bold')\n",
        "plt.xlabel(\"LLMs\", fontsize=20, weight='bold')\n",
        "plt.ylabel(\"Score\", fontsize=16)\n",
        "plt.xticks(fontsize=16, weight='bold')\n",
        "plt.yticks(fontsize=14)\n",
        "plt.ylim(0, 6)\n",
        "\n",
        "# Custom legend\n",
        "handles, labels = plt.gca().get_legend_handles_labels()\n",
        "by_label = dict(zip(labels, handles))\n",
        "plt.legend(by_label.values(), by_label.keys(), bbox_to_anchor=(1.02, 1), loc=\"upper left\", fontsize=12, title=\"Legend\")\n",
        "\n",
        "plt.tight_layout()\n",
        "plt.show()"
      ],
      "metadata": {
        "id": "3Uus-RyLNkVa"
      },
      "execution_count": null,
      "outputs": []
    },
    {
      "cell_type": "code",
      "source": [
        "import pandas as pd\n",
        "import matplotlib.pyplot as plt\n",
        "\n",
        "# Load the Excel file\n",
        "file_path = \"Chamdiem_Trang_0621.xlsx\"  # <-- đổi lại nếu tên file khác\n",
        "df = pd.read_excel(file_path, sheet_name=\"Sheet1\", header=1)\n",
        "\n",
        "# Map LLM codes to names\n",
        "llm_name_map = {\n",
        "    1: \"ChatGPT-4o\",\n",
        "    2: \"Copilot Pro\",\n",
        "    3: \"Claude Sonnet 4\",\n",
        "    4: \"Grok 3\",\n",
        "    5: \"Deepseek\"\n",
        "}\n",
        "df['LLM'] = df['Unnamed: 1'].astype(int).map(llm_name_map)\n",
        "\n",
        "# Tính điểm trung bình giữa 2 người đánh giá\n",
        "df['Bloom_mean'] = df[['Bloom', 'Bloom.1']].mean(axis=1)\n",
        "\n",
        "# Chọn riêng dữ liệu cho mức \"Remember\"\n",
        "remember_df = df[df['Unnamed: 2'] == \"Remember\"]\n",
        "\n",
        "# Lấy danh sách LLM và dữ liệu để vẽ\n",
        "llms = remember_df['LLM'].unique()\n",
        "data_to_plot = [remember_df[remember_df['LLM'] == llm]['Bloom_mean'].dropna() for llm in llms]\n",
        "\n",
        "# Màu pastel kiểu Set3\n",
        "colors = ['#8dd3c7', '#ffffb3', '#bebada', '#fb8072', '#80b1d3']\n",
        "\n",
        "# Vẽ boxplot bằng matplotlib\n",
        "fig, ax = plt.subplots(figsize=(10, 6))\n",
        "box = ax.boxplot(\n",
        "    data_to_plot,\n",
        "    patch_artist=True,\n",
        "    showmeans=True,\n",
        "    showfliers=False,  # Ẩn outliers\n",
        "    meanprops={\"marker\": \"D\", \"markerfacecolor\": \"black\", \"markeredgecolor\": \"black\"}\n",
        ")\n",
        "\n",
        "# Tô màu hộp\n",
        "for patch, color in zip(box['boxes'], colors):\n",
        "    patch.set_facecolor(color)\n",
        "\n",
        "# Định dạng biểu đồ\n",
        "ax.set_xticklabels(llms, fontsize=13)\n",
        "ax.set_title('Mean Bloom Score by LLM for Bloom Level: Remember', fontsize=16)\n",
        "ax.set_ylabel('Mean Bloom Score', fontsize=20)\n",
        "ax.set_xlabel('LLM', fontsize=20)\n",
        "ax.set_ylim(1, 6)\n",
        "ax.grid(True, linestyle='--', alpha=0.5)\n",
        "plt.tight_layout()\n",
        "plt.show()"
      ],
      "metadata": {
        "id": "wRkMm8FtUPJp"
      },
      "execution_count": null,
      "outputs": []
    },
    {
      "cell_type": "code",
      "source": [
        "import pandas as pd\n",
        "import matplotlib.pyplot as plt\n",
        "\n",
        "# Load the Excel file\n",
        "file_path = \"Chamdiem_Trang_0621.xlsx\"  # Change if needed\n",
        "df = pd.read_excel(file_path, sheet_name=\"Sheet1\", header=1)\n",
        "\n",
        "# Map LLM codes to full names\n",
        "llm_name_map = {\n",
        "    1: \"ChatGPT-4o\",\n",
        "    2: \"Copilot Pro\",\n",
        "    3: \"Claude Sonnet 4\",\n",
        "    4: \"Grok 3\",\n",
        "    5: \"Deepseek\"\n",
        "}\n",
        "df['LLM'] = df['Unnamed: 1'].astype(int).map(llm_name_map)\n",
        "\n",
        "# Compute average Bloom score between two evaluators\n",
        "df['Bloom_mean'] = df[['Bloom', 'Bloom.1']].mean(axis=1)\n",
        "\n",
        "# Filter for the \"Understanding\" Bloom level\n",
        "understand_df = df[df['Unnamed: 2'] == \"Understanding\"]\n",
        "\n",
        "# Prepare data for plotting\n",
        "llms = understand_df['LLM'].unique()\n",
        "data_to_plot = [understand_df[understand_df['LLM'] == llm]['Bloom_mean'].dropna() for llm in llms]\n",
        "\n",
        "# Define Set3-like pastel colors\n",
        "colors = ['#8dd3c7', '#ffffb3', '#bebada', '#fb8072', '#80b1d3']\n",
        "\n",
        "# Create the plot\n",
        "fig, ax = plt.subplots(figsize=(10, 6))\n",
        "box = ax.boxplot(\n",
        "    data_to_plot,\n",
        "    patch_artist=True,\n",
        "    showmeans=True,\n",
        "    showfliers=False,  # Hide outliers\n",
        "    meanprops={\"marker\": \"D\", \"markerfacecolor\": \"black\", \"markeredgecolor\": \"black\"}\n",
        ")\n",
        "\n",
        "# Apply color to each box\n",
        "for patch, color in zip(box['boxes'], colors):\n",
        "    patch.set_facecolor(color)\n",
        "\n",
        "# Format the chart\n",
        "ax.set_xticklabels(llms, fontsize=14, fontweight='bold')  # Bigger and bolded LLM names\n",
        "ax.set_title('Mean Bloom Score by LLM for Bloom Level: Understanding', fontsize=16)\n",
        "ax.set_ylabel('Mean Bloom Score', fontsize=14)\n",
        "ax.set_xlabel('LLM', fontsize=14)\n",
        "ax.set_ylim(1, 6)\n",
        "ax.grid(True, linestyle='--', alpha=0.5)\n",
        "plt.tight_layout()\n",
        "plt.show()"
      ],
      "metadata": {
        "id": "Zee1MsvHVvHO"
      },
      "execution_count": null,
      "outputs": []
    },
    {
      "cell_type": "code",
      "source": [
        "import pandas as pd\n",
        "import matplotlib.pyplot as plt\n",
        "\n",
        "# Load the Excel file\n",
        "file_path = \"Chamdiem_Trang_0621.xlsx\"  # Update if needed\n",
        "df = pd.read_excel(file_path, sheet_name=\"Sheet1\", header=1)\n",
        "\n",
        "# Map LLM codes to names\n",
        "llm_name_map = {\n",
        "    1: \"ChatGPT-4o\",\n",
        "    2: \"Copilot Pro\",\n",
        "    3: \"Claude Sonnet 4\",\n",
        "    4: \"Grok 3\",\n",
        "    5: \"Deepseek\"\n",
        "}\n",
        "df['LLM'] = df['Unnamed: 1'].astype(int).map(llm_name_map)\n",
        "\n",
        "# Calculate mean Bloom score from two evaluators\n",
        "df['Bloom_mean'] = df[['Bloom', 'Bloom.1']].mean(axis=1)\n",
        "\n",
        "# Filter for the \"Applying\" Bloom level\n",
        "apply_df = df[df['Unnamed: 2'] == \"Applying\"]\n",
        "\n",
        "# Get LLM list and group data\n",
        "llms = apply_df['LLM'].unique()\n",
        "data_to_plot = [apply_df[apply_df['LLM'] == llm]['Bloom_mean'].dropna() for llm in llms]\n",
        "\n",
        "# Define Set3-style pastel colors\n",
        "colors = ['#8dd3c7', '#ffffb3', '#bebada', '#fb8072', '#80b1d3']\n",
        "\n",
        "# Create the plot\n",
        "fig, ax = plt.subplots(figsize=(10, 6))\n",
        "box = ax.boxplot(\n",
        "    data_to_plot,\n",
        "    patch_artist=True,\n",
        "    showmeans=True,\n",
        "    showfliers=False,  # Hide outliers\n",
        "    meanprops={\"marker\": \"D\", \"markerfacecolor\": \"black\", \"markeredgecolor\": \"black\"}\n",
        ")\n",
        "\n",
        "# Apply colors\n",
        "for patch, color in zip(box['boxes'], colors):\n",
        "    patch.set_facecolor(color)\n",
        "\n",
        "# Format plot\n",
        "ax.set_xticklabels(llms, fontsize=14, fontweight='bold')\n",
        "ax.set_title('Mean Bloom Score by LLM for Bloom Level: Applying', fontsize=16)\n",
        "ax.set_ylabel('Mean Bloom Score', fontsize=14)\n",
        "ax.set_xlabel('LLM', fontsize=14)\n",
        "ax.set_ylim(1, 6)\n",
        "ax.grid(True, linestyle='--', alpha=0.5)\n",
        "plt.tight_layout()\n",
        "plt.show()"
      ],
      "metadata": {
        "id": "ZasNOEKjWlxi"
      },
      "execution_count": null,
      "outputs": []
    },
    {
      "cell_type": "code",
      "source": [
        "import pandas as pd\n",
        "import matplotlib.pyplot as plt\n",
        "\n",
        "# Load the Excel file\n",
        "file_path = \"Chamdiem_Trang_0621.xlsx\"  # Change this if your file name is different\n",
        "df = pd.read_excel(file_path, sheet_name=\"Sheet1\", header=1)\n",
        "\n",
        "# Map numeric LLM codes to full model names\n",
        "llm_name_map = {\n",
        "    1: \"ChatGPT-4o\",\n",
        "    2: \"Copilot Pro\",\n",
        "    3: \"Claude Sonnet 4\",\n",
        "    4: \"Grok 3\",\n",
        "    5: \"Deepseek\"\n",
        "}\n",
        "df['LLM'] = df['Unnamed: 1'].astype(int).map(llm_name_map)\n",
        "\n",
        "# Calculate average Bloom score from two evaluators\n",
        "df['Bloom_mean'] = df[['Bloom', 'Bloom.1']].mean(axis=1)\n",
        "\n",
        "# Filter for the \"Analysing\" Bloom level (British spelling)\n",
        "analyse_df = df[df['Unnamed: 2'] == \"Analysing\"]\n",
        "\n",
        "# Prepare data per LLM\n",
        "llms = analyse_df['LLM'].unique()\n",
        "data_to_plot = [analyse_df[analyse_df['LLM'] == llm]['Bloom_mean'].dropna() for llm in llms]\n",
        "\n",
        "# Set3 pastel-style color palette\n",
        "colors = ['#8dd3c7', '#ffffb3', '#bebada', '#fb8072', '#80b1d3']\n",
        "\n",
        "# Create the plot\n",
        "fig, ax = plt.subplots(figsize=(10, 6))\n",
        "box = ax.boxplot(\n",
        "    data_to_plot,\n",
        "    patch_artist=True,\n",
        "    showmeans=True,\n",
        "    showfliers=False,  # Hide outliers\n",
        "    meanprops={\"marker\": \"D\", \"markerfacecolor\": \"black\", \"markeredgecolor\": \"black\"}\n",
        ")\n",
        "\n",
        "# Color each box\n",
        "for patch, color in zip(box['boxes'], colors):\n",
        "    patch.set_facecolor(color)\n",
        "\n",
        "# Final formatting\n",
        "ax.set_xticklabels(llms, fontsize=14, fontweight='bold')\n",
        "ax.set_title('Mean Bloom Score by LLM for Bloom Level: Analysing', fontsize=16)\n",
        "ax.set_ylabel('Mean Bloom Score', fontsize=14)\n",
        "ax.set_xlabel('LLM', fontsize=14)\n",
        "ax.set_ylim(1, 6)\n",
        "ax.grid(True, linestyle='--', alpha=0.5)\n",
        "plt.tight_layout()\n",
        "plt.show()"
      ],
      "metadata": {
        "id": "ejCXkxX8W1ax"
      },
      "execution_count": null,
      "outputs": []
    },
    {
      "cell_type": "code",
      "source": [
        "import pandas as pd\n",
        "import matplotlib.pyplot as plt\n",
        "\n",
        "# Load the Excel file\n",
        "file_path = \"Chamdiem_Trang_0621.xlsx\"  # Adjust if necessary\n",
        "df = pd.read_excel(file_path, sheet_name=\"Sheet1\", header=1)\n",
        "\n",
        "# Map numeric LLM codes to descriptive names\n",
        "llm_name_map = {\n",
        "    1: \"ChatGPT-4o\",\n",
        "    2: \"Copilot Pro\",\n",
        "    3: \"Claude Sonnet 4\",\n",
        "    4: \"Grok 3\",\n",
        "    5: \"Deepseek\"\n",
        "}\n",
        "df['LLM'] = df['Unnamed: 1'].astype(int).map(llm_name_map)\n",
        "\n",
        "# Compute the average Bloom score from two evaluators\n",
        "df['Bloom_mean'] = df[['Bloom', 'Bloom.1']].mean(axis=1)\n",
        "\n",
        "# Filter for the \"Creating/Evaluation\" Bloom level\n",
        "create_eval_df = df[df['Unnamed: 2'] == \"Creating/Evaluation\"]\n",
        "\n",
        "# Prepare data to plot per LLM\n",
        "llms = create_eval_df['LLM'].unique()\n",
        "data_to_plot = [create_eval_df[create_eval_df['LLM'] == llm]['Bloom_mean'].dropna() for llm in llms]\n",
        "\n",
        "# Set3-style pastel colors\n",
        "colors = ['#8dd3c7', '#ffffb3', '#bebada', '#fb8072', '#80b1d3']\n",
        "\n",
        "# Plotting\n",
        "fig, ax = plt.subplots(figsize=(10, 6))\n",
        "box = ax.boxplot(\n",
        "    data_to_plot,\n",
        "    patch_artist=True,\n",
        "    showmeans=True,\n",
        "    showfliers=False,  # Hide outliers\n",
        "    meanprops={\"marker\": \"D\", \"markerfacecolor\": \"black\", \"markeredgecolor\": \"black\"}\n",
        ")\n",
        "\n",
        "# Apply colors to boxes\n",
        "for patch, color in zip(box['boxes'], colors):\n",
        "    patch.set_facecolor(color)\n",
        "\n",
        "# Format chart\n",
        "ax.set_xticklabels(llms, fontsize=14, fontweight='bold')\n",
        "ax.set_title('Mean Bloom Score by LLM for Bloom Level: Creating/Evaluation', fontsize=16)\n",
        "ax.set_ylabel('Mean Bloom Score', fontsize=14)\n",
        "ax.set_xlabel('LLM', fontsize=14)\n",
        "ax.set_ylim(1, 6)\n",
        "ax.grid(True, linestyle='--', alpha=0.5)\n",
        "plt.tight_layout()\n",
        "plt.show()"
      ],
      "metadata": {
        "id": "_iyQrsTBXYNe"
      },
      "execution_count": null,
      "outputs": []
    },
    {
      "cell_type": "code",
      "source": [
        "import pandas as pd\n",
        "import matplotlib.pyplot as plt\n",
        "import seaborn as sns\n",
        "\n",
        "# Load the Excel file\n",
        "file_path = \"Chamdiem_Trang_0621.xlsx\"  # Update path if needed\n",
        "df = pd.read_excel(file_path, sheet_name=\"Sheet1\", header=1)\n",
        "\n",
        "# Rename for clarity\n",
        "df['LLM'] = df['Unnamed: 1'].astype(int).map({\n",
        "    1: \"ChatGPT-4o\",\n",
        "    2: \"Copilot Pro\",\n",
        "    3: \"Claude Sonnet 4\",\n",
        "    4: \"Grok 3\",\n",
        "    5: \"Deepseek\"\n",
        "})\n",
        "df['Bloom_Level'] = df['Unnamed: 2']\n",
        "df['Bloom_mean'] = df[['Bloom', 'Bloom.1']].mean(axis=1)\n",
        "\n",
        "# Group and pivot to get LLMs as columns and Bloom levels as rows\n",
        "heatmap_data = df.groupby(['LLM', 'Bloom_Level'])['Bloom_mean'].mean().unstack()\n",
        "\n",
        "# Reorder Bloom levels bottom-up (taxonomy order reversed)\n",
        "bloom_order = ['Remember', 'Understanding', 'Applying', 'Analysing', 'Creating/Evaluation']\n",
        "heatmap_data_ordered = heatmap_data.T.reindex(bloom_order[::-1])  # Bottom-up\n",
        "\n",
        "# Plot heatmap\n",
        "plt.figure(figsize=(14, 6))  # Make x-axis longer\n",
        "sns.heatmap(\n",
        "    heatmap_data_ordered,\n",
        "    annot=True,\n",
        "    cmap='YlGnBu',\n",
        "    fmt=\".2f\",\n",
        "    linewidths=0.5,\n",
        "    linecolor='gray',\n",
        "    cbar_kws={\"label\": \"Mean Bloom Score\"}\n",
        ")\n",
        "\n",
        "# Formatting\n",
        "plt.title(\"Mean Bloom Scores per Bloom Level across LLMs\", fontsize=18, pad=20)\n",
        "plt.xlabel(\"LLM\", fontsize=16, labelpad=10)\n",
        "plt.ylabel(\"Bloom's Cognitive Level\", fontsize=16)\n",
        "plt.xticks(fontsize=14, fontweight='bold')\n",
        "plt.yticks(fontsize=13, rotation=0)\n",
        "plt.tight_layout()\n",
        "plt.show()"
      ],
      "metadata": {
        "id": "nYTz1z4Kc3e9"
      },
      "execution_count": null,
      "outputs": []
    }
  ]
}
```
