# Supplementary material for: Evaluating cognitive depth of AI-generated multiple-choice questions with Bloom’s Taxonomy
Source: PLoS One. 2026 Feb 27;21(2):e0341317. doi: 10.1371/journal.pone.0341317 (PMC12948114; doi:10.1371/journal.pone.0341317)
Supplement: Attachment — Submitted filename: Response to reviewers.docx [file pone.0341317.s005.docx]

**Manuscript PONE-D-25-36938**

**Response to Reviewers**

Dear Editorial Board
*PLOS ONE*,

Thank you for giving us the opportunity to submit a revised draft of the manuscript “Evaluating Cognitive Depth in AI-Generated Multiple-Choice Questions: A Comparative Study of Modern Large Language Models Using Bloom’s Taxonomy” for publication in the *PLOS ONE*. We appreciate the time and effort you and the reviewers dedicated to providing feedback on our manuscript and are grateful for the insightful comments and valuable improvements to our paper.

In response to the reviewer’s inquiry regarding ethical approval, we would like to clarify that the study was approved by the Institutional Ethics Committee of [University Name]. However, this information has been temporarily anonymized in the manuscript to ensure a double-blind review process. We will update the ethical approval statement with the full institutional details and approval number upon acceptance.

We have incorporated most of the suggestions made by the reviewers. Those changes are highlighted within the manuscript in red. Please see below, in blue, for a point-by-point response to the reviewers’ comments and concerns. All page numbers refer to the revised manuscript file with tracked changes.

Reviewers' Comments to the Authors:

# #1 Reviewer

It is a great pleasure to have reviewed this paper, “Evaluating Cognitive Depth in AI-Generated Multiple-Choice Questions: A Comparative Study of Modern Large Language Models Using Bloom’s Taxonomy”. The study has numerous merits, but for this review, I will unravel some grey areas that, after implementing the correction, will further strengthen the quality of the paper.

- **Title and Abstract**
- Title (L1–3): The title is informative and clearly indicates scope, methods, and framework. However, it is a little long. You might shorten to: “Evaluating Cognitive Depth of AI-Generated Multiple-Choice Questions with Bloom’s Taxonomy”. The “Short Title” already captures this succinctly.

**Author response**: Thank you for your helpful suggestion. We have revised the title to make it more concise while ensuring it remains informative.
Please refer to the updated title in lines 1–2, page 1 of the revised manuscript.

- Abstract (L4–30): The abstract is structured but slightly repetitive.

The “Introduction” sentence could be condensed into one line (“While LLMs are used to generate medical and dental MCQs, their alignment with Bloom’s Taxonomy remains unexplored.”).

**Author response**: Thank you for your advice. We have condensed the introductory sentence of the abstract into a single, concise statement to improve clarity and reduce redundancy.
Please refer to the changes in lines 5-6, page 1 of the revised manuscript.

The “Materials and Methods” section is clear, but you should specify that each model generated 60 MCQs (total 300) upfront for transparency.

**Author response**: Thank you for your suggestion. We have added clarification that a total of 300 MCQs were generated across all LLMs (60 per model) to enhance transparency.
Please refer to the revisions in line 10, page 1 of the manuscript.

In the “Results,” provide exact p-values where significant, not just p < 0.05.

**Author response**: Thank you for your advice. We have specified the exact p-values for significant results in the Results section of the abstract.
Please refer to the changes in lines 22-25, page 2 of the revised manuscript.

In “Conclusions,” avoid subjective phrasing (“superior performance”) and instead state “Claude Sonnet 4 achieved the highest alignment at higher-order levels.”

**Author response**: Thank you for your valuable feedback. We have rephrased the conclusion to remove subjective wording and emphasize the factual outcome, stating that Claude Sonnet 4 achieved the highest alignment at higher-order levels.
Please see the updated text in lines 27-28, page 2 of the revised manuscript.

- **Introduction**
- Opening (L31–38): The rationale for MCQs is well stated, but the phrasing is verbose. Consider shortening: “MCQs provide a cost-effective way to assess knowledge, comprehension, and problem-solving in large cohorts, but creating high-quality, cognitively targeted items requires expertise and resources.”

**Author response**: Thank you for your valuable comment. We have revised the opening paragraph to make the explanation of MCQs more concise and less wordy.

Please see the updated text in lines 30-32, page 2 of the revised manuscript.

- Bloom’s Taxonomy (L39–43): Good explanation. Ensure consistent naming (“Analyze,” “Evaluate/Create”) throughout the manuscript. Consider referencing updated frameworks (e.g., Anderson & Krathwohl’s 2001 revision).

**Author response:** Thank you for your insightful suggestion. We have updated the manuscript to reference the Anderson and Krathwohl (2001) revision of Bloom’s Taxonomy. In addition, we have ensured consistent use of the terms **“**Analyze**”** and **“**Evaluate/Create**”** throughout the text.
Please refer to the changes in lines 36, page 2, and reference numbered 8 of the revised manuscript.

- AI and LLM background (L44–49): Strong context, but some citations are older (e.g., GPT-3 work). Since you are submitting in 2025, reference more recent reviews (e.g., Küchemann et al., 2025).

**Author response:** Thank you for your helpful comment. We agree that the rapid evolution of LLMs warrants the inclusion of more recent literature. Accordingly, we have replaced older citation that include GPT-3 (Luo D. et al) with up-to-date studies (e.g., Küchemann et al., 2025 and Alohali KI et al., 2025) to better reflect the current state of LLM development.
Please refer to the updates in lines 41-48, page 3 and reference numbered 19 of the revised manuscript.

- Research gap (L50–57): Well identified, but should emphasize novelty: “To date, no comparative evaluation of state-of-the-art LLMs has assessed their ability to generate MCQs across Bloom’s levels.”

**Author response:** Thank you for your suggestion. We have incorporated your proposed wording to emphasize the novelty and comparative focus of our study.
Please see the revised text in lines 46-48, page 3 of the manuscript.

- **Methods**
- Design (L60–67): Clear statement of cross-sectional comparative design. Consider explicitly noting IRB exemption if no human subjects were involved.

**Author response:** Thank you for pointing this out. The study was approved by the Institutional Ethics Committee of [University Name], but the information was temporarily hidden to maintain anonymity during the review process. We have now included the ethical approval statement in the revised manuscript as “[University Name], Approval No. XXX” for anonymity purposes, and will update it with the full details upon acceptance.

Please refer to the updates in lines 56-58, page 3 of the revised manuscript**.**

- LLM Selection (L68–76): Strong justification, listing release dates and subscription status. Add citations or URLs to each model’s technical report for transparency.

**Author response:** Thank you for your valuable suggestion. We have added the official URLs for each LLM to enhance transparency and traceability.

Please refer to the updates made in lines 68-71 on page 4 of the revised manuscript.

- Sample Size (L77–81): You correctly reference G*Power. Specify the test family (e.g., Kruskal–Wallis) and effect size convention. Good decision to use 60 items per LLM.

**Author response:** Thank you

- Source Material and Prompt (L82–99): Very detailed, which is a strength.

**Author response:** Thank you

- However, Table 1 is overly long—condense to 1–2 illustrative examples. Avoid full explanations that overwhelm the reader.

**Author response:** Thank you for your suggestion. We have shortened Table 1 to 2 examples of questions generated by ChatGPT-4o.

Please see the change in the updated Table 1 of the revised manuscript (pages 6-7)

- Evaluation (L105–115): Well structured with two independent raters. Provide rater demographics (teaching years, academic level) for credibility.

**Author response:** Thank you for your suggestion. We have added details on the raters’ academic qualifications and teaching experience to enhance the credibility of the evaluation process.
Please refer to the revisions made in the Methods section (page 7, lines 104-106).

- Statistics (L117–123): You describe use of SPSS and Python. Good practice. Re-word sentence at L121–122 for clarity: “Inter-model comparisons were conducted using the Kruskal–Wallis test, followed by Bonferroni-corrected post hoc tests.”

**Author response:** Thank you for your suggestion. We have rephrased the description of the statistical analysis to improve clarity, specifying that inter-model comparisons were conducted using the Kruskal–Wallis test followed by Bonferroni-corrected post hoc analyses.
Please refer to the revisions made in the Methods section (page 8, lines 116-117).

- **Results**
- Inter-rater reliability (L126–133): Good reporting of weighted kappa values. Consider citing Yilmaz & Demirhan (2023) for weighted kappa interpretation.

**Author response:** Thank you for your comment. We have acknowledged and cited Yilmaz & Demirhan (2023) to support the interpretation of weighted kappa values used in our original manuscript.
Please refer to the updated text in the revised manuscript (page 9, lines 122-124, citation number 23).

- Model comparison (L135–139): Table 3 is comprehensive. Ensure alignment in table formatting (some rows misaligned). Report effect sizes (η² or rank-biserial) alongside p-values.

**Author response:** Thank you for your valuable feedback. We have revised Table 3 to improve its formatting and alignment for clearer presentation. In addition, we have included the corresponding effect size (η²) alongside the p-values where applicable to enhance the depth of statistical interpretation.
Please refer to the updated Table 3 and the accompanying text on pages 9-12 of the revised manuscript.

- Figures (L148–179): Figures 2–4 are useful but captions are too brief. Add interpretive context (e.g., “Claude Sonnet 4 scored highest at Applying, Analyzing, and Evaluating/Creating levels”).

**Author response:** Thank you for your helpful suggestion. We have expanded the captions for Figures 2–4 to include brief interpretive context, highlighting key findings such as the relative performance of each LLM across Bloom’s cognitive levels.
Please refer to the revised figure captions on page 11, lines 147-148; page 12, lines 162-165; pages 13-14, lines 180-183 of the manuscript.

- Text results (L166–174): The within-model analysis is strong, but rephrase sentences for conciseness. Example: “For ChatGPT-4o, Remembering items scored higher than Analyzing and Evaluating/Creating (p < 0.01).”

**Author response:** Thank you for your suggestion. We have rephrased the within-model results section to improve conciseness and clarity while maintaining accuracy in statistical reporting.
Please refer to the revised text on page 13, lines 170-172 of the updated manuscript.

- **Discussion**
- Strengths of study (L180–186): Clear articulation of novelty. Good acknowledgment that this is the first systematic LLM-Bloom evaluation.

**Author response:** Thank you

- Interpretation (L187–218): Insightful comparison, but avoid over-interpreting. For example, stating “Claude Sonnet 4 consistently outperformed…” should be softened: “Claude Sonnet 4 achieved higher alignment at higher levels.”

**Author response:** Thank you for your suggestion. We have revised the discussion to moderate the interpretive tone and avoid overstating model performance. The phrasing has been adjusted to emphasize that “Claude Sonnet 4 achieved higher alignment at higher cognitive levels” rather than suggesting consistent outperformance.

Please refer to the updated text in lines 195-196, page 14 of the revised manuscript.

- Integration with prior work (L193–200): Nicely cites medical/dental MCQ research. Ensure consistent referencing style (some journal abbreviations missing).

**Author response:** Thank you for your comment. We have carefully reviewed and revised the reference list to ensure a consistent citation format, including correction of missing journal abbreviations where applicable.

Please refer to the References section of the revised manuscript.

- Mechanisms (L219–229): Well-reasoned discussion on architecture, training data, and fine-tuning. Ensure references to Vaswani (2017), Fedus (2022), and Kaplan (2020) are up to date.

**Author response:** Thank you for your comment. We have verified and updated the references related to model architecture, training data, and fine-tuning mechanisms to ensure accuracy and relevance. Specifically, the citations to Vaswani et al. (2017) and Kaplan et al. (2020) have been reviewed and replaced or supplemented with the most recent and appropriate sources where applicable.

Please refer to the References section of the revised manuscript.

- Educational implications (L231–247): Strong discussion of healthcare education implications. Add practical takeaway: “Educators may selectively use Claude or Copilot for advanced assessments, while ChatGPT-4o may be more suitable for foundational testing.”

**Author response:** Thank you for your insightful suggestion. We have added a practical takeaway highlighting the applicability of our findings in educational settings, noting that educators may consider using Claude for advanced assessments and ChatGPT-4o for foundational-level testing.
Please refer to the revisions made in lines 253-256, page 17 of the revised manuscript.

- Limitations (L252–260): Limitations are acknowledged (subjectivity of raters, Bloom focus only, exclusion of multimodal tasks). Strengthen by explicitly noting: (1) only dental anatomy domain, (2) prompt design may bias outputs.

**Author response:** Thank you for your valuable feedback. We have strengthened the Limitations section by explicitly addressing two additional constraints: (1) the study’s focus on the dental anatomy domain, which may limit generalizability to other fields, and (2) the potential influence of prompt design on the quality and cognitive alignment of LLM-generated outputs.
Please refer to the updated text in lines 279-283, page 18 of the revised manuscript.

- **Conclusions**
- Concluding sentence (L263–269): Strong, but rephrase for precision: “Claude Sonnet 4 demonstrated the highest ability to generate higher-order questions, while all LLMs performed well at lower levels.” Avoid aspirational phrases (“fully realized”).

**Author response:** Thank you for your constructive feedback. We have revised the concluding sentence to enhance precision and remove aspirational language. The updated version now reads:
“Claude Sonnet 4 demonstrated the strongest ability to generate higher-order questions, while all evaluated LLMs performed well at lower cognitive levels.”
Please refer to page 19, lines 293-295 of the revised manuscript.

- **References**
- Strengths: Includes foundational MCQ literature and recent 2023–2025 LLM evaluations.

**Author response:** Thank you!

- Weaknesses: Some inconsistencies (missing italics for journal names, inconsistent use of initials). Ensure uniform adherence to PLOS ONE reference style. Verify DOI formatting (should be lowercase “doi:” not capitalized).

**Author response:** Thank you for pointing this out. We have revised the reference list to ensure full compliance with the journal’s formatting guidelines, as outlined on the PLOS ONE webpage. Specifically, we corrected journal title italics, standardized author initials, and ensured consistent citation formatting.

Example of a reference format from the PLOS ONE website:

Hou WR, Hou YL, Wu GF, Song Y, Su XL, Sun B, et al. cDNA, genomic sequence cloning and overexpression of ribosomal protein gene L9 (rpL9) of the giant panda (Ailuropoda melanoleuca). Genet Mol Res. 2011;10:1576–1588.

Please refer to the updated Reference section in the revised manuscript.

**Overall Assessment**

***Strengths:***

Novel and timely evaluation of LLMs in education.

Strong methodological rigor (sample size, inter-rater reliability, nonparametric statistics).

Practical implications for dental/medical education.

**Author response:** Thank you

***Weaknesses:***

Verbosity in Abstract, Introduction, and Methods.

Overly detailed Table 1 examples.

Need for clearer limitations (domain, prompt, subjective scoring).

Figures and tables require more explanatory captions.

References need style consistency.

***Recommendations:***

1. Shorten Abstract and Introduction for concision.

2. Reduce Table 1 to 1–2 examples; move others to Supplementary.

3. Add effect sizes to results.

4. Rephrase strong claims in Discussion and Conclusion.

5. Expand Limitations section explicitly.

6. Revise reference formatting per PLOS ONE guidelines.

**Author response:** Thank you for all your valuable feedback. We have revised the manuscript to reduce verbosity, condensed Table 1 to a single representative example, and expanded the Limitations section to address domain specificity, subjective scoring, and dependence on prompt design. Additionally, we have enhanced the figure captions for clarity and ensured consistency throughout the reference list.

**#2 Reviewer :**

- This is an interesting research which has the potential of having direct impact on professional teaching similar course. I would like the authors to address of the thoughts I had as I read the manuscript in the hope to making it better for readers.

Author response: Thank you for your thoughtful comments and constructive suggestions, which have helped us improve the clarity and readability of our manuscript. Our detailed responses are as follows:

- Provide some citation to back the statement in line 50-52

**Author response:** Thank you for your suggestion. We have added supporting citations from Ahmed WM et al. (2024), Kim HS et al. (2025), and Law AK et al. (2025) to strengthen the statement in lines 50–52 of the original manuscript.
Please refer to the updated text in lines 44-45, page 3, references numbered 14, 10, 21 of the revised manuscript.

- It will be more helpful to have the figures at the appropriate location they are mentioned in the manuscript instead of after reference. Not seeing those figures break flow of reading.

**Author response:** Thank you for your helpful feedback. We have repositioned all figures to appear immediately after their first mention in the text to improve readability and maintain a smoother flow for the reader.

Please refer to Figures 1-4 in the revised manuscript.

- In table 3, I am not sure what the values in the parenthesis represents. Some clarification would be helpful to readers.

**Author response:** Thank you for pointing this out. The values in parentheses represent the interquartile range (IQR) of the data. We have clarified this in the caption of Table 3 to ensure transparency and ease of interpretation.
Please refer to the updated caption of Table 3 in line 132, page 10 of the revised manuscript.

- I was hoping so see some kind of quick highlight table showing the performance based on free and paid LLMs as stated in line 248. Is there any takeaway from that?

**Author response**: Thank you for your valuable suggestion. Although our study did not specifically focus on comparing the advantages and disadvantages of free versus paid LLMs, it is evident that models with both subscription and free-access options—such as ChatGPT, Claude Sonnet, and Copilot—typically offer higher usage limits and faster response times in their paid versions. Nonetheless, several free-access LLMs, particularly Claude Sonnet 4 (free-access version) and Grok 3, demonstrated comparable or even superior performance at higher-order cognitive levels of Bloom’s Taxonomy. We have now included a summary table (Table 4) highlighting the performance differences between free and paid LLMs, along with the key takeaway.

Please refer to Table 4 and the corresponding revisions in lines 265-268, page 17 of the updated manuscript.

- Can you provide some justification for the use of Kruskal-Wallis? Line 121

**Author response**: Thank you for your comment. The Kruskal–Wallis test was selected for multiple-group comparisons because the data were ordinal in nature, making this non-parametric test suitable for comparing mean ranks across models.
We have added a clear justification for this choice in the Methods section of the revised manuscript.
Please refer to the updated text in page 8, line 116.

- I am wondering if the use of LLM will reduce the usual time used for developing MCQ? Of course it would potentially reduce time it takes to write but what about need for editing? Would there be need for proficiency in prompt engineering? Could that be a roadblock for potential use?

**Author response**: Thank you for this insightful comment. We agree that one of the key advantages of LLM-assisted MCQ generation is the potential to substantially reduce the time required for initial item development. However, we have acknowledged in the revised Discussion that although LLMs can accelerate the drafting process, a degree of expert review and editing remains essential to ensure accuracy, content validity, and alignment with intended learning objectives.

We have also noted that effective use of LLMs depends on basic proficiency in prompt design, which could initially pose a barrier for some educators. Nonetheless, as interfaces become more user-friendly and models continue to improve, this limitation is likely to diminish over time.

Please refer to the updated text in page 17, lines 256-263 of the revised manuscript.

**#3 Editors**

The authors present an important and timely contribution to understanding how large language models (LLMs) generate multiple-choice questions aligned with Bloom’s Taxonomy. However, several major revisions are required to enhance clarity, methodological transparency, and structural organisation.

- The Abstract, Introduction, and portions of the Methods section are overly verbose, containing repetitive explanations that obscure key points. These sections should be tightened for precision and readability.

**Author response**: Thank you for your comment. We have revised the Abstract, Introduction, and relevant sections of the Methods to enhance clarity, conciseness, and overall readability by removing redundant and repetitive content.

Please refer to the updated text in lines 5-6, 10, 22-28 pages 1-2; 30-32, page 2, 116-117, page 8 of the revised manuscript.

The title may also be shortened to “Evaluating Cognitive Depth of AI-Generated Multiple-Choice Questions with Bloom’s Taxonomy” for conciseness and impact.

**Author response:** Thank you for your helpful suggestion. We have revised the title to make it more concise while retaining clarity and relevance to the study’s scope.

Please refer to the updated text in lines 1–2, page 1 of the revised manuscript.

- Figures should be repositioned to appear near their first in-text mention, as their current placement after the References disrupts reading flow.

**Author response**: Thank you for this suggestion. We have repositioned all figures to appear immediately after their first in-text mention to improve the logical flow and readability of the manuscript.
Please refer to Figures 1-4 in the revised manuscript.

In Table 3, the meaning of the values in parentheses should be clearly defined, while Table 1 should be reduced to one or two representative examples, with additional items moved to supplementary materials.

**Author response**: Thank you for your helpful advice. We have clarified in the Table 3 caption that the values in parentheses represent the interquartile range (IQR). Additionally, Table 1 has been condensed to include two representative examples, with remaining examples moved to the Supplementary Materials to maintain brevity.
Please refer to Table 1 and Table 3 captions in the revised manuscript (page 10, line 132).

A short highlight table contrasting the performance of free and paid LLMs, as noted in line 248, would improve interpretability and reader engagement.

**Author response**: Thank you for your valuable suggestion. We have added a summary highlight table (Table 4) presenting the comparative performance of free versus paid LLMs, along with a concise takeaway to enhance interpretability.
Please refer to Table 4 and the corresponding text revisions in page 17, lines 265-268 of the revised manuscript.
